# Enhancing Methane Aromatization Performance by Reducing the Particle Size of Molybdenum Oxide

**DOI:** 10.3390/nano10101991

**Published:** 2020-10-09

**Authors:** Jing Hu, Jinghai Liu, Jinglin Liu, Yangyang Li, Peihe Li, Yin Wang, Jingqi Guan, Qiubin Kan

**Affiliations:** 1 College of Chemistry and Materials Science, Inner Mongolia University for Nationalities, Tongliao 028000, China; hwhujing@163.com (J.H.); Jhliu2008@sinano.ac.cn (J.L.); Jlliu2000@126.com (J.L.); 15114714255@163.com (Y.L.); 2 Inner Mongolia Key Laboratory of Carbon Nanomaterials, Nano Innovation Institute (NII), Inner Mongolia University for Nationalities, Tongliao 028000, China; phli2018@foxmail.com (P.L.); Wy19890703@126.com (Y.W.); 3 Institute of Physical Chemistry, College of Chemistry, Jilin University, Changchun 130023, China

**Keywords:** molybdenum oxide, methane aromatization, HMCM-49, aromatics

## Abstract

Efficient use of natural gas to produce aromatics is an attractive subject; the process requires catalysts that possess high-performance active sites to activate stable C–H bonds. Here, we report a facile synthetic strategy to modify HMCM-49 with small molybdenum oxide nanoparticles. Due to the higher sublimability of nano-MoO_3_ particles than commercial MoO_3_, they more easily enter into the channels of HMCM-49 and associate with Brønsted acid sites to form active MoC_x_-type species under calcination and reaction conditions. Compared with commercial MoO_3_ modified MCM-49, nano-MoO_3_ modified MCM-49 exhibits higher methane conversion (13.2%), higher aromatics yield (9.1%), and better stability for the methane aromatization reaction.

## 1. Introduction

The ongoing discovery of cheap natural gas has stimulated increased interest in converting the main component, methane, to higher value-added fuels and chemicals [1]. The commercial processes through which methane is converted into a mixture of carbon monoxide and hydrogen (syngas) include steam reforming, dry reforming, auto-thermal reforming, and partial reforming [2,3]. However, converting methane into liquid fuel through an indirect catalytic method is challenging. A direct, economical process of methane dehydro-aromatization (MDA) could better use these resources. MDA is therefore a promising method that has received extensive attention in the last few decades [4].

The MDA of molybdenum-modified HZSM-5 has attracted researchers’ attention since Wang et al. first reported on it in 1993 [5]. The HZSM-5 zeolite was considered as one of the best carriers [6,7] and molybdenum was the optimal loading component for MDA [8,9,10]. However, CH_4_ possesses high C–H bond strength (434 kJ·mol^−1^), resulting in an extremely low equilibrium for methane conversion (~12% at 700 °C), high coke deposition, and severe deactivation caused by Mo loss at high temperatures [11]. Seeking catalysts with high catalytic activity and stability for this reaction therefore remains a significant challenge.

Mo-based MDA catalysts are usually prepared by mechanical mixing, leading to the dispersion of Mo(VI)-oxo species on the outside surface of the zeolite and inside the microporous channel of the zeolite through high-temperature treatment. The Mo species react with acidic protons to form [MoO_2_]^2+^ monomers and [Mo_2_O_5_]^2+^ dimers [12]. The dispersion of MoO_x_ species has significant influence on the Mo content in the zeolite channel. A portion of Mo species is converted to oxide aggregates at high Mo loadings [13]. Mo-oxo species are reduced to MoC_x_O_y_ species during the induction period of the reaction and then fully carburized into a MoC_x_-type phase during the reaction procedure, which is located in the micropore channel and at the external surface of the zeolite [9]. It is universally acknowledged that the MoC_x_-type phase is the active center for methane non-oxidative aromatization, as revealed by XPS [14], CH_4_-TPSR (temperature programmed surface reaction) [15], ^95^Mo MAS (magic-angle spinning) NMR [16], and EXAFS (extended x-ray absorption fine structure) [17].

Many strategies have been adopted to reduce carbon deposition on active sites by improving the dispersion of Mo species. Better Mo species dispersion leads to less coking. Vollmer et al. demonstrated that zeolite topology has a strong influence on MDA activity and selectivity [18]. Zeolites with a 10-MR structure that closely resemble ZSM-5 show high MDA performance due to comparable channel dimensions. Zeolites (e.g., MCM-22 [19], MCM-49 [20], and TNU-9 [21]) exhibit similar or better catalytic activity and selectivity with benzene than the ZSM-5 zeolite. Zeolites with MWW topology possess three complex independent channel systems: 12-ring cups (half supercages 7.1 × 7.1 × 9.1 Å) on the external surface, an interlayer with 12-ring supercages (7.1 Å outside diameter × 18.2 Å height) through 10-ring opening windows (4.1 × 5.4 Å and 4.1 × 5.9 Å), and an intralayer with 2D sinusoidal 10-ring pores (4.1 × 5.4 Å). The dispersion of active centers in different pore systems of MWW zeolites, which are solid acidic catalysts, depends on the accessibility of the acid sites, diffusion lengths, and reaction spaces for the transition state, which will affect the catalytic performance of MDA [22].

With the rapid development of nanotechnology, the nanostructure of transition-metal oxides has attracted great attention due to its exceptional potential for catalytic applications [23,24,25]. Molybdenum trioxide (MoO_3_) is a promising material for energy storage [26], field emission [27], catalysis [28,29,30,31,32], etc. For example, subnano-MoO_3_ clusters coordinating with amino-functionalized silica exhibit excellent catalytic performance in oxidative desulfurization [33]. A single-site Mo-containing nanosized ZSM-5 zeolite with homogenous dispersion of Mo atoms shows superior catalytic activity and stability [28]. In this study, we investigated the activity of nano-MoO_3_-modified HMCM-49 in MDA. We found that nano-MoO_3_-doped HMCM-49 showed higher methane conversion, higher benzene yield, and better stability than commercial MoO_3_-modified HMCM-49.

## 2. Materials and Methods 

### 2.1. Catalyst Preparation

#### 2.1.1. Synthesis of MoO_3_ Nanoparticles

MoO_3_ nanoparticles were synthesized as follows: 2 g of ammonium heptamolybdate tetrahydrate (≥99.0%, Sigma-Aldrich, Saint Louis, MO, USA) was dissolved in a 50 mL mixture of H_2_O and ethanol. An amount of 0.5 g of polyethylene glycol (PEG-2000, Alfa Aeser, Royston, UK) was then added to the mixture and stirred for 2 h. After the pH value was adjusted to 4.5 with an HNO_3_ (65.0~68.0%, analytic reagent, Damao Chemical Reagent Factory, Tianjin, China) solution, the mixture was transferred to a Teflon-lined stainless autoclave and heated at 110 °C for 24 h. The sediment was filtrated, washed with H_2_O, and dried overnight in a vacuum oven at 80 °C to obtain nano-MoO_3_, which was designated as MoO_3_(N).

#### 2.1.2. Synthesis of HMCM-49 Zeolites

The MCM-49 zeolites were prepared with a hydrothermal synthesis method using hexamethyleneimine (HMI, 99%, Sigma-Aldrich, Saint Louis, MI, USA) as the template and silica gel (AS-40, SiO_2_: 40 wt %, Sigma-Aldrich, Saint Louis, USA) as the silica source [34]. The original slurry was obtained by stirring a mixture of sodium aluminate (NaAlO_2_, Al_2_O_3_: > 45%, Sigma-Aldrich, Saint Louis, USA), sodium hydroxide (≥98%, Sigma-Aldrich, Saint Louis, MI, USA), HMI, silica gel, and water. The molar ratio of the gel composition was SiO_2_: 0.02, Al_2_O_3_: 0.35, and HMI: 25 H_2_O. The reaction mixture was crystallized at 170 °C with a rotating rate of 60 rpm for 3 d. The resulting solid was filtrated, washed with distilled water, and dried overnight in an oven at 60 °C. The MCM-49 precursor was dispersed with a 30 wt % H_2_O_2_ solution (analytical grade, Damao Chemical Reagent Factory, Tianjin, China) in a round-bottomed flask at 90 °C for 12 h and then filtered and dried under ambient conditions. The HMCM-49 zeolites were obtained twice after an ion exchange with 1 M NH_4_NO_3_ (analytical grade, Damao Chemical Reagent Factory, Tianjin, China) aqueous solution at 90 °C for 6 h followed by calcination in a muffle furnace at 500 °C for 8 h.

#### 2.1.3. Synthesis of Mo/HMCM-49

Mo-species-modified HMCM-49 was prepared as follows: commercial MoO_3_ (≥99.5%, Sigma-Aldrich, Saint Louis, MI, USA, marked as MoO_3_(C)) and MoO_3_(N) were mechanically mixed with HMCM-49 to prepare Mo/HMCM-49 containing 2–8 wt % MoO_3_, which was then calcined in air at 500 °C for 5 h to obtain Mo(C)-HMCM-49 and Mo(N)-HMCM-49.

### 2.2. Catalyst Characterization 

The samples were measured with an XRD-6000 diffractometer with Cu-Kα radiation (Shimadzu Corporation, Kyoto, Japan). The textural properties of the catalysts were measured by N_2_ physisorption at –196 °C on ASAP-2000 equipment (Micromeritics, Atlanta, GA, USA). The SEM image was obtained with an FESEM XL-30 field emission scanning electron microscope (Hitachi Corporation, Tokyo, Japan). EDS mappings were performed with an FEI Tecnai G2 F30 STWIN field emission transmission electron microscope (FEI corporation, Eindhoven, Netherlands). NH_3_-TPD was carried out to estimate the acid strength distribution measured by the desorption of ammonia from 100 to 600 °C at a heating rate of 10 °C/min. IR spectroscopy of pyridine adsorption was also performed to measure the acid content. All samples were activated in a form of self-supporting wafers at 200 °C under vacuum for 30 min prior to pyridine adsorption. After fully adsorbing pyridine for 1 h at 100 °C, the sample was outgassed in situ under vacuum at 100 °C for 1 h to physically remove the adsorbed pyridine. Thermogravimetric analysis was conducted with a Shimadzu DTG-60 (Shimadzu Corporation, Kyoto, Japan). The samples were heated to 800 °C at a rate of 5 °C/min in a dry-air atmosphere.

### 2.3. Catalytic Test

The MoO_3_/HMCM-49 catalysts were evaluated for methane non-oxidative aromatization in a continuous flow fixed-bed quartz reactor with 1 cm ID. The reaction was carried out by a mass flow controller at 700 °C with a flow of CH_4_/N_2_ 92.5%/7.5% (15 mL/min). The production was analyzed using an online gas chromatograph equipped with a 6 m × 3 mm HayeSep D 80/100 column (Shimadzu Corporation, Kyoto, Japan) to split H_2_, N_2_, CO, CO_2_, CH_4_, C_2_H_4_, and C_2_H_6_, and a CBP1-M50-025 quartz capillary column (Shimadzu Corporation, Kyoto, Japan) to separate benzene, toluene, and naphthalene. Helium was used as the carrier gas and the N_2_ internal standard method of analysis was used to calculate methane conversion and hydrocarbon product selectivity on the basis of carbon mass balance [35].

## 3. Result and Discussion

### 3.1. MDA Tests

**Figure 1:**
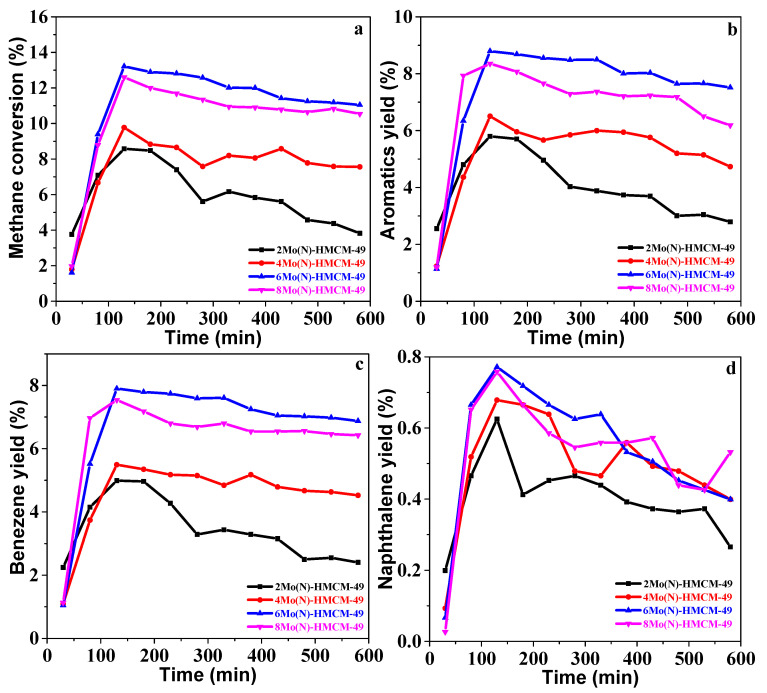
(**a**) Methane conversion, (**b**) Aromatics yield, (**c**) Benzene yield, and (**d**) Naphthalene yield over 2Mo(N)-HMCM-49 (■), 4Mo(N)-HMCM-49 (●), 6Mo(N)-HMCM-49 (▲), and 8Mo(N)-MCM-49 (▼). Reaction conditions: T (temperature) = 700 °C, P (pressure) = 1 atm, GHSV(gas firing hourly space velocity) = 1500 h^−1^.

It has been reported that non-modified HZSM-5 and Mo/NaZSM-5 catalysts exhibit poor MDA activity due to a lack of active or acidic sites [5,36,37]. Although commercial MoO_3_-modified HMCM-49 showed good MDA activity, stability was poor [20,38]. Figure 1 depicts the tendency of methane to convert and yield to aromatics, benzene, and naphthalene as a function of time in the stream for MDA over the Mo(N)-HMCM-49 catalysts. MDA can generally be divided into two stages. Initially, the Mo-oxo species was carburized to MoC_x_ after introducing CH_4_, and methane conversion and aromatics yield reached quasi-steady values [13,39]. During the second stage, methane conversion and yield reduced gradually, which is ascribed to deactivation by remarkable coke deposition as well as metal carbide species sintering and detaching from MCM-49. Methane conversion over 2Mo(N)-HMCM-49, 4Mo(N)-HMCM-49, 6Mo(N)-HMCM-49, and 8Mo(N)-HMCM-49 reached a maximum of 8.5%, 9.7%, 13.2%, and 12.5%, respectively, and the corresponding aromatics product was 5.8%, 6.5%, 9.1%, and 8.3%. Methane conversion over the catalysts gradually decreased with increasing reaction time. At 580 min, methane conversion over 6Mo(N)-HMCM-49 was 11.0%, which was higher than over 2Mo(N)-HMCM-49 (3.8%), 4Mo(N)-HMCM-49 (7.6%), and 8Mo(N)-HMCM-49 (10.5%). MCM-49 possesses a set of 12 MR cages connected through 10 MR windows and a set of 10 MR channels. The two pore systems were responsible for the generation of benzene and aromatics [22]. Heavy hydrocarbons and coke deposits were mainly generated over Mo carbides located on the external surface of the zeolite, and thus the methane conversion and aromatics yield have a downward trend due to carbon deposition in the reaction process [40]. Relevant studies have confirmed that Brønsted acid centers can combine well with Mo species, but excessive Brønsted acid centers are conducive to the generation of aromatic carbon deposition, preventing the pore channels of the molecular sieve from deactivating the catalyst [41]. As a result, when the loading of MoO_3_(N) is 6%, the catalyst exhibits optimal performance. We found that MoO_3_(N) can effectively transfer to the pore channels of MCM-49 and interact with Brønsted acid centers to reasonably adjust the distribution of acidic sites, generating more effective active sites to achieve preeminent catalytic performance.

**Figure 2:**
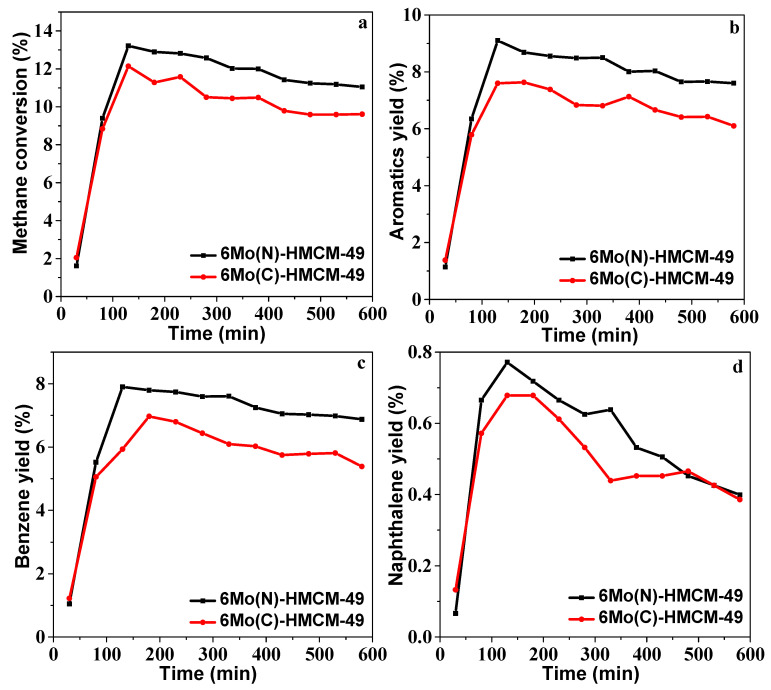
(**a**) Methane conversion, (**b**) Aromatics yield, (**c**) Benzene yield, and (**d**) Naphthalene yield over 6Mo(C)-MCM-49 and 6Mo(N)-MCM-49. Reaction conditions: T = 700 °C, P = 1 atm, GHSV = 1500 h^−1^.

**Figure 3:**
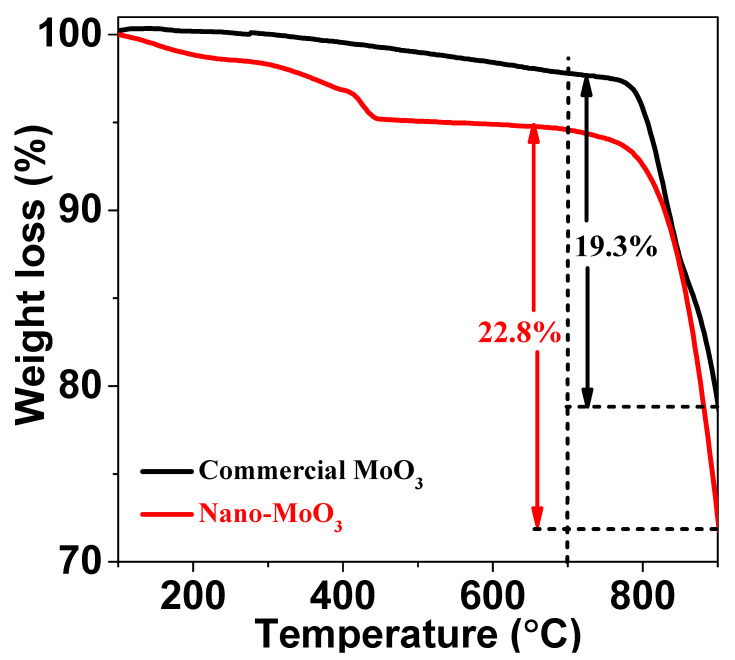
Thermogravimetric analysis of commercial MoO_3_ and ano-MoO_3_.

The methane conversion, selectivity, and aromatics yield over 6Mo(C)-HMCM-49 and 6Mo(N)-HMCM-49 in MDA within 580 min are shown in Figure 2 and Table 1. 6Mo(C)-HMCM-49 was deactivated after reacting for 130 min, in agreement with a prior report [34]. Methane conversion over 6Mo(N)-HMCM-49 reached a maximal value of 13.2%, higher than that over 6Mo(C)-HMCM-49 (12.1%). When the reaction reached 580 min, the methane conversion over 6Mo(N)-HMCM-49 was 11.0%, much higher than over 6Mo(C)-HMCM-49 (9.6%). We calculated that 6Mo(N)-HMCM-49 can maintain a higher retention of maximal activity than 6Mo(C)-HMCM-49 at 580 min (83.3% vs. 79.3%), indicating better stability with the former. Moreover, the selectivity of benzene over 6Mo(N)-HMCM-49 is higher than over 6Mo(C)-HMCM-49. At 130 min, a 9.1% aromatics yield is achieved over 6Mo(N)-HMCM-49, which is higher than over 6Mo(C)-HMCM-49 (7.6%). The increased stability may be due to the effective distribution of α-MoC_x_ active centers. The status of the catalyst precursor may play a key role in the activity [39]. The metal precursor loading has a variety of configurations on the zeolite, which leads to complicated and broadened signals for most spectroscopic techniques. As discussed below, the pyridine FTIR and NH3-TPD results showed that the acidity of HMCM-49 is altered by nano-MoO_3_ modification. Furthermore, thermogravimetric analysis showed that nano-MoO_3_ sublimes more easily than commercial MoO_3_. As depicted in Figure 3, the weight loss of nano-MoO_3_ between 700 and 900 °C was 22.8%, which is higher than that of commercial MoO_3_ (19.3%), demonstrating easier sublimation of the former. As a consequence, nano-MoO_3_ has better dispersion in the pore channels of MCM-49 and interacts well with Brønsted acid centers to generate more active MoC_x_-type centers for MDA, showing higher activity and stability.

### 3.2. XRD Characterization

**Figure 4:**
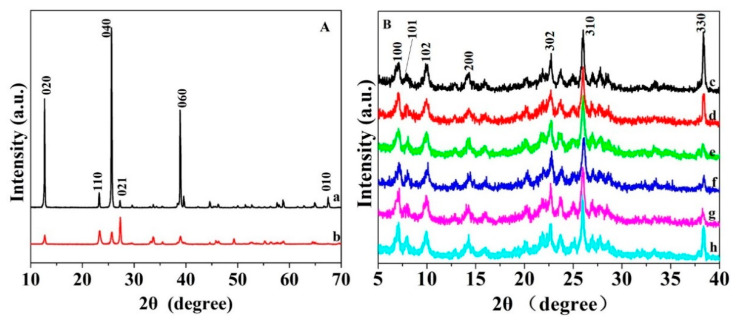
XRD patterns in (**A**): (**a**) MoO_3_(C) and (**b**) MoO_3_(N) and (**B**): (**c**) HMCM-49, (**d**) 2Mo(N)-HMCM-49, (**e**) 4Mo(N)-HMCM-49, (**f**) 6Mo(N)-HMCM-49, (**g**) 8Mo(N)-HMCM-49, and (**h**) 6Mo(C)-HMCM-49.

To reveal the crystallinity structure of nano-MoO_3_ and Mo(N)-HMCM-49, XRD characterization was performed. As shown in Figure 4A, the sharp diffraction peaks of nano-MoO_3_ at 2θ = 12.9° and 27.4° confirm a highly crystalline α-MoO_3_ orthorhombic phase. The diffraction peak intensity of (021) planes was quite high, indicating a crystal orientation along (001) [42]. The crystallite size of MoO_3_(N) was estimated to be about 22 nm. The XRD patterns of HMCM-49 and Mo species-doped HMCM-49 are shown in Figure 4B. The peaks at 2θ of 7.1°, 8.0°, 10°, 14.2°, 22°, 26°, and 37.9° for MCM-49 were due to 100, 101, 102, 200, 302, 310, and 330 crystal planes, respectively [16]. All catalysts exhibited characteristic peaks of MCM-49. Nevertheless, the peak intensity of MoO_3_(N)-modified MCM-49 decreased compared to pristine HMCM-49, suggesting that the diffusion of MoO_3_ in the pores causes the loss of crystallinity. No obvious peaks due to MoO_3_ were observed even at high Mo loadings, implying that MoO_3_ nanoparticles were well dispersed on the surface and in the channels of HMCM-49. Moreover, as compared to the 6Mo(C)-HMCM-49 catalyst (Figure 4h), the 6Mo(N)-HMCM-49 sample showed lower crystallinity (Figure 4f), especially for the 310- and 330-crystal planes, which confirms that more Mo-species migrated to HMCM-49 channels.

### 3.3. SEM Characterization

**Figure 5:**
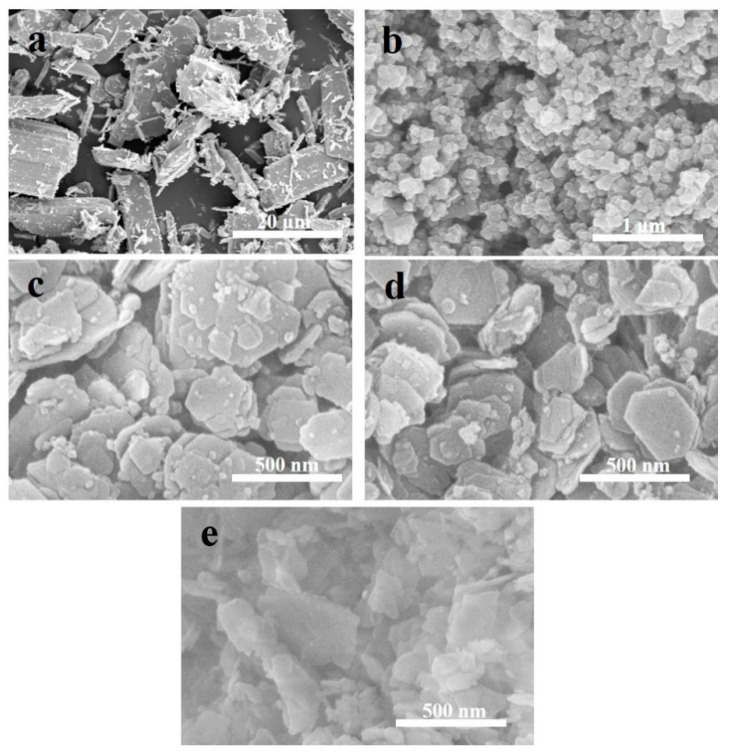
SEM images of (**a**) commercial MoO_3_, (**b**) MoO_3_(N), (**c**) HMCM-49, (**d**) 6Mo(N)-HMCM-49, and (**e**) 6Mo(C)-HMCM-49.

The morphology of commercial MoO_3_, MoO_3_(N), HMCM-49, and 6Mo(N)-HMCM-49 was analyzed by SEM. As shown in Figure 5, the particle size of commercial MoO_3_ is larger than 1 μm, whereas the particle size of MoO_3_(N) is less than 50 nm. In addition, we clearly observed that HMCM-49 has a platelet-like shape, in accordance with the literature [43]. MCM-49 has a length of less than 500 nm and a thickness of less than 50 nm. The morphology of MCM-49 did not significantly change after nano-MoO_3_ modification. Some small crystallites were found on the surface of 6Mo(N)-HMCM-49, which was attributed to the cracking of large MCM-49 crystallites by grinding. The surface of MCM-49 modified with commercial MoO_3_ presented larger particles, which may be attributed to more commercial MoO_3_ located on the external surface of the zeolite. The above results further evidence that nano-MoO_3_ more easily sublimates and diffuses into the zeolite channels.

### 3.4. TEM Characterization

**Figure 6:**
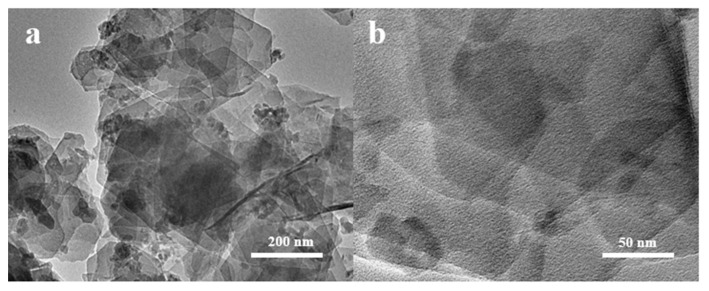
(**a**,**b**) TEM images of 6Mo(N)-HMCM-49 with different magnifications.

**Figure 7:**
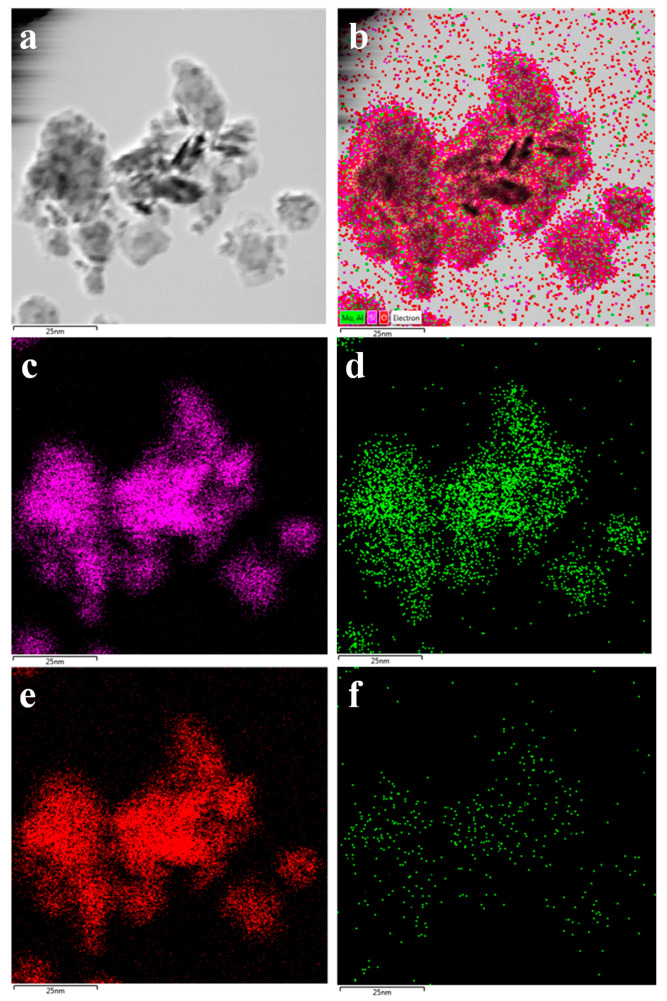
TEM image of 6Mo(N)-HMCM-49 used in the EDS mapping test (**a**), and the corresponding EDS mapping of various elements (**b**), Si (**c**), Al (**d**), O (**e**), and Mo (**f**).

**Figure 8:**
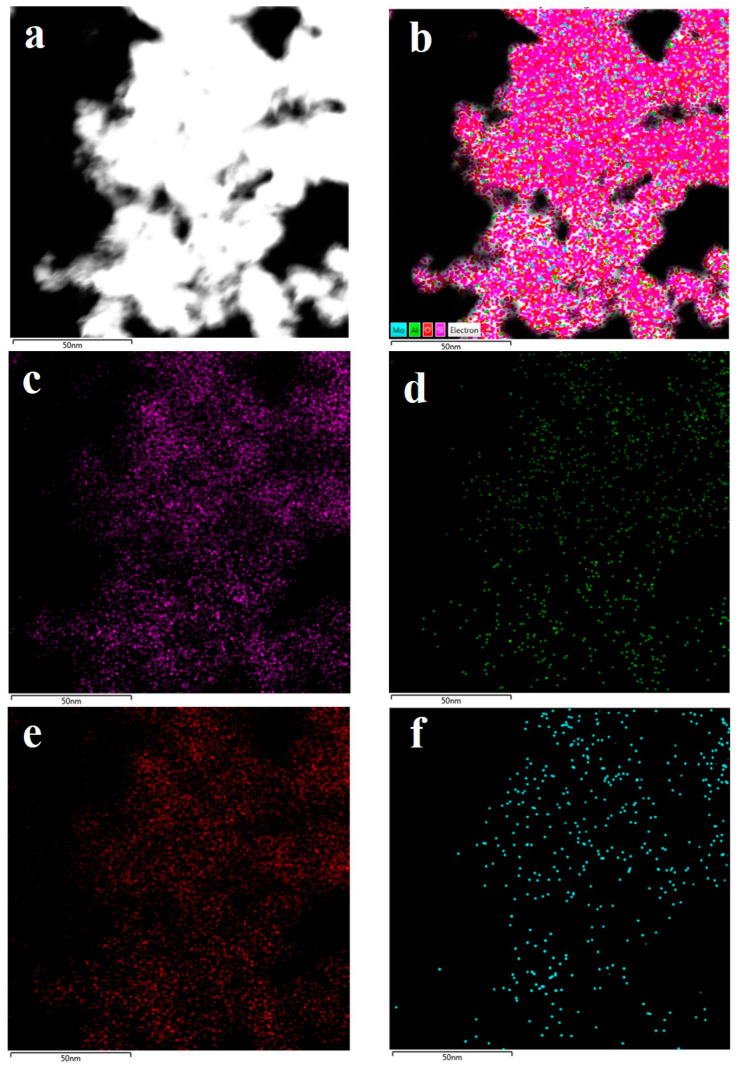
TEM image of 6Mo(C)-HMCM-49 used in the EDS mapping test (**a**) and the corresponding EDS mapping of various elements (**b**), Si (**c**), Al (**d**), O (**e**), and Mo (**f**).

The morphology of 6Mo(N)-HMCM-49 was further characterized by TEM. As illustrated in Figure 6, a thin plate-like shape was typically observed for the MCM-49-based materials, in agreement with the SEM results. To identify the dispersion of Mo species in HMCM-49, EDS analysis was performed. As exhibited in Figure 7, 6Mo(N)-HMCM-49 contained Si, Al, O, and Mo elements, which were uniformly dispersed. Comparatively, the Mo species exhibited worse distribution in 6Mo(C)-HMCM-49 (Figure 8).

### 3.5. N_2_ Adsorption-Desorption Characterization

**Figure 9:**
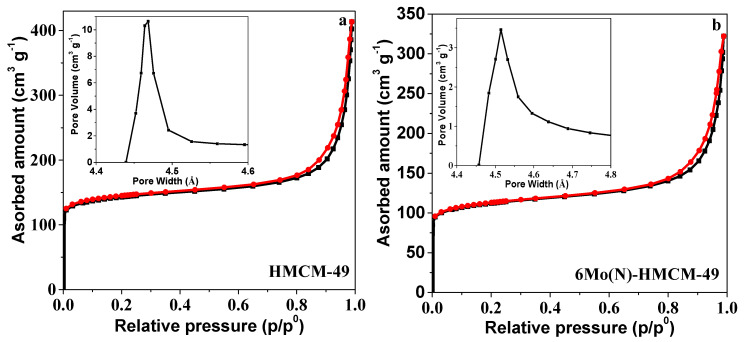
Nitrogen adsorption-desorption isotherms and pore size distributions of (**a**) HMCM-49 and (**b**) 6Mo(N)-MCM-49.

The textural properties of HMCM-49 and 6Mo(N)-MCM-49 were revealed by N_2_ adsorption-desorption isotherms and pore size distributions extracted from the adsorption branch of isotherms (Figure 9 and Table 2). The isotherm of MCM-49 is typical isotherm I, indicating a microporous zeolite. A steep line at relatively low pressure is associated with capillary condensation in micropores of the HMCM-49 zeolite. A narrow hysteresis loop can be seen for MCM-49 at P/P^0^ = 0.6–1.0, indicating the presence of slit-mesopore platelet particles most likely derived from the aggregation of stratified particles. The micropore area and volume of Mo/HMCM-49 gradually decreased with a continually increasing content of nano-MoO_3_ loadings, indicating that the content of MoO_3_(N) has a specific effect on the structural parameters of HMCM-49. The specific surface area decreased from 482 to 379 cm^2^/g and micropore volume decreased from 0.16 to 0.12 cm^3^/g after loading 6 wt % MoO_3_(N). These results imply that Mo species migrate into the zeolitic channels, where the Mo-oxo species can anchor on the external/internal surface of the zeolite and lead to a decrease in specific surface area and pore volume [44]. In addition, the specific surface area of 6Mo(N)-MCM-49 was smaller than that of 6Mo(C)-MCM-49, implying that nano-MoO_3_ shows better dispersion in the micropore channel of the HMCM-49 zeolite due to easier sublimation of nano-MoO_3_ than commercial MoO_3_.

### 3.6. Acidity Characterization

**Figure 10:**
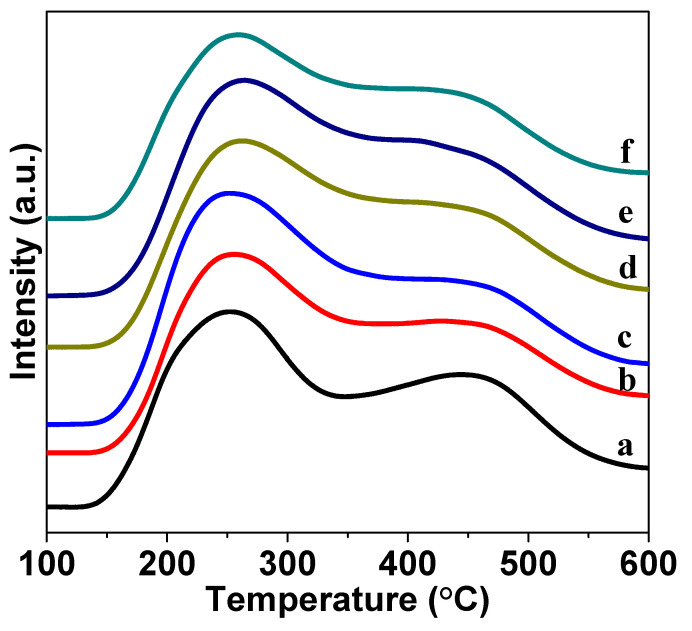
NH_3_-TPD profiles of (**a**) HMCM-49, (**b**) 2Mo(N)-HMCM-49, (**c**) 4Mo(N)-HMCM-49, (**d**) 6Mo(N)-HMCM-49, (**e**) 8Mo(N)-HMCM-49, and (**f**) 6Mo(C)-HMCM-49.

**Figure 11:**
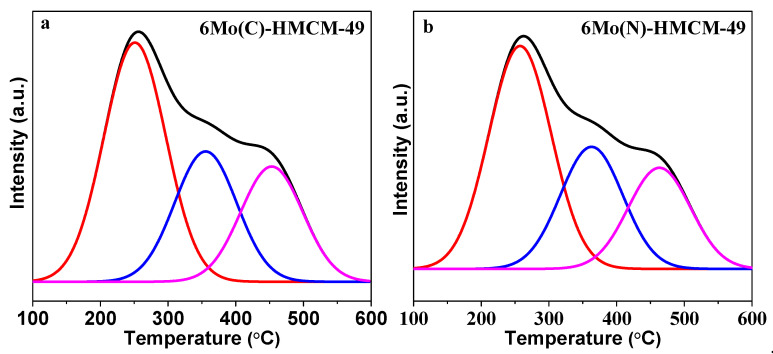
NH_3_-TPD profiles of the 6Mo(C)-HMCM-49 and 6Mo(N)-HMCM-49 catalysts after fitting curves.(**a**) 6Mo(C)-HMCM-49,(**b**) 6Mo(N)-HMCM-49

The acidity of Mo/HMCM-49 was analyzed by NH_3_-TPD. As shown in Figure 10, different desorption temperatures of NH_3_-TPD can reflect acid strength. The low temperature peak in the range of 200–300 °C is ascribed to the decomposition of physically adsorbed NH_3_ on the catalyst, while the high temperature peak between 400 and 500 °C is attributed to the decomposition of NH_3_ adsorbed on Brønsted acid sites [45]. The NH_3_-TPD profiles exhibited a new peak at ca. 360 °C after fitting curves, as shown in Figure 11, which is related to the desorption of the NH_3_ adsorbed on exchangeable protonic sites. After loading MoO_3_, the peak associated with Brønsted acid sites became weak, indicating that a portion of strong acid sites interacts with the Mo species [43]. In addition, the acid strength of the catalyst gradually decreased with the increase in nano-MoO_3_, further confirming that MoO_3_(N) reacts with the acidic sites of HMCM-49 [46]. The relative amounts of acid sites in HMCM-49 and Mo-HMCM-49 were calculated by Gaussian fitting. As shown in Table 3, the Brønsted acid strength of MoO_3_(N)-HMCM-49 decreased more significantly than 6Mo(C)-HMCM-49 when the same amount of MoO_3_ was loaded, suggesting that more MoO_3_(N) species migrate into the channels of the zeolite and bind with Brønsted acid sites.

**Figure 12:**
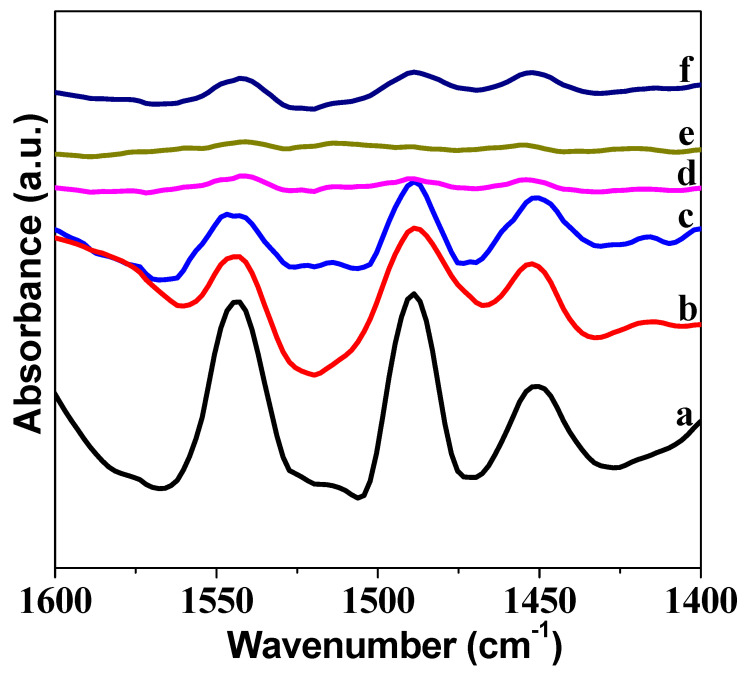
FTIR spectra in the pyridine region after desorption of the base at 100 °C: (**a**) HMCM-49, (**b**) 2Mo(N)-HMCM-49, (**c**) 4Mo(N)-HMCM-49, (**d**) 6Mo(N)-HMCM-49, (**e**) 8Mo(N)-HMCM-49, and (**f**) 6Mo(C)-HMCM-49.

The acidity of Mo/HMCM-49 was further characterized by pyridine FTIR measurements. As exhibited in Figure 12, the adsorption of pyridine on Brønsted acid sites was about 1540 cm^−1^, whereas the adsorption of pyridine on Lewis acid sites corresponded to a peak of about 1450 cm^−1^ [47]. The peak of about 1489 cm^−1^ is related to pyridine interacting with medium-strength acid sites. The acid strength of Brønsted acid sites and Lewis acid sites clearly decreased with the increasing content of nano-MoO_3_ loadings, which is consistent with the TPD results above. It has been reported that the formation of highly dispersed Mo species associated with the internal Brønsted acid sites is crucial for methane non-aromatization [18,48]. The smaller the size of the MoO_3_ particles, the better the dispersion of Mo species in the zeolite channels.

### 3.7. Thermogravimetric Analysis of the Catalysts After Reactions

**Figure 13:**
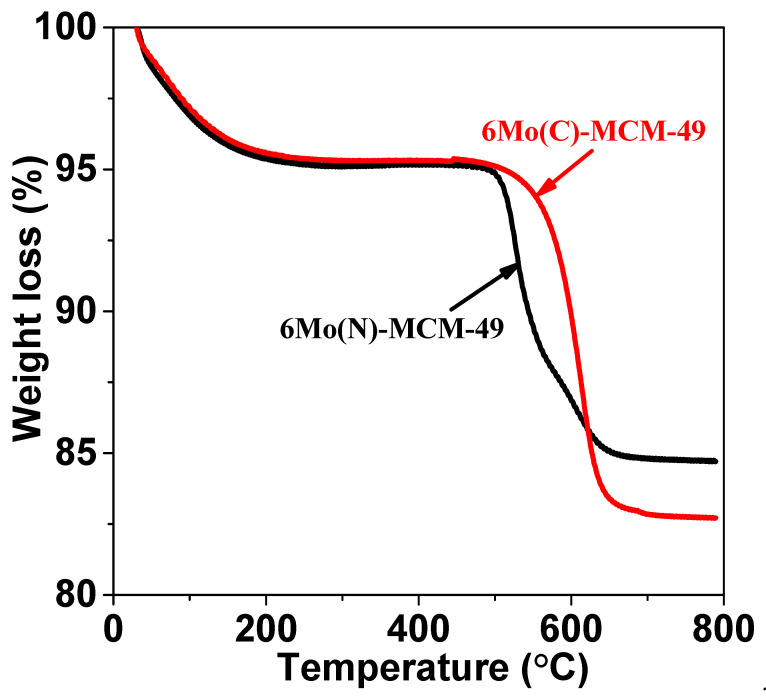
Thermogravimetric analysis of 6Mo(C)-HMCM-49 and 6Mo(N)-HMCM-49 after reacting for 580 min. The catalysts after reaction were analyzed by TGA.

As depicted in Figure 13, weight loss below 200 °C is associated with absorbed water. Weight loss at 350–450 °C is due to the oxidation of Mo carbide species. The noteworthy weight loss between 400 and 800 °C is assigned to the burn-off of carbon deposition formed on the zeolite [49]. Benzene forms mainly in the pore channels of the zeolites, while naphthalene forms mainly on the surface and orifices of the zeolites [50]. Carbonaceous deposition in the zeolite channels may prompt the loss of partial active centers and block the zeolite channels, thus causing catalyst deactivation. During the reaction induction period, carbon deposition reached a maximal value of selectivity over 6Mo(C)-HMCM-49 and 6Mo(N)-HMCM-49 of 37.4% and 30.4%, respectively. As the reaction continued, the selectivity of carbon deposition decreased. The weightlessness of 6Mo(C)-HMCM-49 and 6Mo(N)-HMCM-49 after reacting for 580 min was about 12.6% and 9.6%, respectively, proving that 6Mo(N)-HMCM-49 shows better resistance to carbon deposition than 6Mo(C)-HMCM-49. According to pyridine FTIR and NH_3_-TPD results, more active MoC_x_-type sites are generated in micropores of nano-MoO_3_-doped HMCM-49, which favors benzene for improvement of selectivity and results in better catalytic performance.

## 4. Conclusions

In summary, we found that nano-MoO_3_-modified MCM-49 showed better MDA performance than commercial MoO_3_-modified MCM-49, achieving 13.2% of methane conversion and 9.1% of aromatics yield. Due to its small size, nano-MoO_3_ can sublimate more easily and diffuse into the channels to react with MCM-49 acid sites and form active MoC_x_-type sites, which can prevent the blocking of pores and improve stability. This study provided a facile strategy to synthesize high-performance and stable catalysts for MDA.

## Figures and Tables

**Table 1:** Catalytic results of methane non-oxidative aromatization over various catalysts.

Catalyst	Time (min)	Conversion (%)	Selectivity (%)				Aromatics Yield
			Benzene	Toluene	Naphthalene	Coke	
6Mo(C)-MCM-49	130	12.1	54.5	4.0	4.1	37.4	7.6
	580	9.6	55.2	4.1	4.1	36.6	6.1
6Mo(N)-MCM-49	130	13.2	59.8	4.5	5.3	30.4	9.1
	580	11.0	61.8	4.5	4.0	29.7	7.7

**Table 2:** Textural properties of HMCM-49, 6Mo(C)-HMCM-49, and HMCM-49 modified with different contents of nano-MoO_3._

Sample	Surface Area (m^2^/g)	Micropore Area (m^2^/g)	Micropore Volume (cm^3^/g)
HMCM-49	482	353	0.16
6Mo(C)-HMCM-49	404	270	0.12
2Mo(N)-HMCM-49	469	337	0.15
4Mo(N)-HMCM-49	404	280	0.13
6Mo(N)-HMCM-49	379	263	0.12
8Mo(N)-HMCM-49	367	275	0.12

**Table 3:** Peak temperature of NH_3_-TPD spectra and the relative amounts of acid sites in HMCM-49 and Mo-HMCM-49.

Samples	Peak (L)		Peak (M)		Peak (H)	
	Peak Temp (°C)	Area Unit	Peak Temp (°C)	Area Unit	Peak Temp (°C)	Area Unit
HMCM-49	228	445			416	627
6Mo(N)-HMCM-49	223	315	277	175	414	325
6Mo(C)-HMCM-49	231	338	287	198	424	412
